# Antibiotic resistance in *Salmonella* spp. isolated from poultry: A global overview

**DOI:** 10.14202/vetworld.2020.2070-2084

**Published:** 2020-10-03

**Authors:** Rafael Enrique Castro-Vargas, María Paula Herrera-Sánchez, Roy Rodríguez-Hernández, Iang Schroniltgen Rondón-Barragán

**Affiliations:** 1 Research Group in Immunology and Pathogenesis, Faculty of Veterinary Medicine and Zootechnics, University of Tolima, Santa Helena Highs, Ibagué, Tolima, Colombia; 2 Poultry Research Group, Faculty of Veterinary Medicine and Zootechnics, University of Tolima, Santa Helena Highs, Ibagué, Tolima, Colombia

**Keywords:** antimicrobial, poultry, resistance, *Salmonella*

## Abstract

*Salmonella enterica* is the most important foodborne pathogen, and it is often associated with the contamination of poultry products. Annually, *Salmonella* causes around 93 million cases of gastroenteritis and 155,000 deaths worldwide. Antimicrobial therapy is the first choice of treatment for this bacterial infection; however, antimicrobial resistance has become a problem due to the misuse of antibiotics both in human medicine and animal production. It has been predicted that by 2050, antibiotic-resistant pathogens will cause around 10 million deaths worldwide, and the WHO has suggested the need to usher in the post-antibiotic era. The purpose of this review is to discuss and update the status of *Salmonella* antibiotic resistance, in particular, its prevalence, serotypes, and antibiotic resistance patterns in response to critical antimicrobials used in human medicine and the poultry industry. Based on our review, the median prevalence values of *Salmonella* in broiler chickens, raw chicken meat, and in eggs and egg-laying hens were 40.5% ( interquartile range [IQR] 11.5-58.2%), 30% (IQR 20-43.5%), and 40% (IQR 14.2-51.5%), respectively. The most common serotype was *Salmonella* Enteritidis, followed by *Salmonella* Typhimurium. The highest antibiotic resistance levels within the poultry production chain were found for nalidixic acid and ampicillin. These findings highlight the need for government entities, poultry researchers, and producers to find ways to reduce the impact of antibiotic use in poultry, focusing especially on active surveillance and finding alternatives to antibiotics.

## Introduction

*Salmonella enterica* subsp. *enterica* is one of the most important foodborne pathogens worldwide and remains the leading cause of infectious gastroenteritis. Cases are often related to the consumption of food of animal origin, mainly poultry products, such as eggs and raw chicken [1-3]. Globally, non-typhoidal *Salmonella* causes around 93 million cases of gastroenteritis and 155 000 deaths each year [4-6]. The disease manifestation depends on the serotype involved, virulence factors, infective dose, and host immunity. Immunocompromised patients, children, and elderly people tend to be more susceptible and suffer more serious clinical symptoms, including sepsis [7-11]. In other cases, the infection can cause a chronic state of asymptomatic carriage in the host [7]. The serotypes *Salmonella* Enteritidis and *Salmonella* Typhimurium are the most frequent causes of salmonellosis in humans [12-14]. However, emerging serotypes such as *Salmonella* Heidelberg, *Salmonella* Javiana, *Salmonella* Infantis, and *Salmonella* Thompson have been reported to infect humans in the United States and are becoming more prevalent in certain segments of the poultry production chain [2,15].

Meanwhile, antimicrobial resistance is another global threat in animal and human medicine. Its dangers lie mainly in the failure to successfully treat patients infected with antibiotic-resistant pathogens and in the high risk of transmission of such resistant pathogens [16]. The development of this resistance is related to the misuse of antibiotics, including their use in animal production systems as growth promoters and their excessive use in clinical treatments [16,17]. This is a great concern because much of the antibiotic-resistant *Salmonella* have been acquired through the consumption of contaminated food of animal origin, resulting in health risk to humans and increasing the cost of health care [4,18,19]. Some authors have predicted that antimicrobial-resistant pathogens will be responsible for 10 million deaths worldwide by the year 2050 [20,21]. In addition, the use of generic antimicrobials in veterinary and human medicine poses a risk because certain strains of bacteria with β- lactamases and/or AmpC β-lactamases have been isolated from food animal products. In addition, extended-spectrum cephalosporin-resistant *Salmonella* has been isolated from poultry. These bacteria have been responsible for failure in human treatments, resulting in a demand for a second line of antibiotics to control infections [22,23]. Different government agencies have encouraged the prudent use of antibiotics in veterinary and human medicine. For example, antimicrobials such as carbapenems, glycopeptides, tigecycline, and third- and fourth-generation cephalosporins have been restricted in their use. Antibiotic resistance in *Salmonella* strains and virulent clones can compromise infection treatment in humans, making it difficult to control the disease, and poses a severe risk to global public health [24]. Thus, the World Health Organization has defined *Salmonella* as a “priority pathogen” and aims to guide and promote research and development into new antibiotics for its treatment [11,25].

This review discusses the worldwide prevalence, serotypes, and antibiotic resistance patterns of *Salmonella* isolates from different segments of the poultry production chain.

## Antibiotic Resistance of *Salmonella* Isolates

Several multidrug-resistant (MDR) *Salmonella* strains have been isolated in beef, pork, and poultry products, and each of these has the potential to spread and generate a global emergency [22,26,27]. In the case of poultry products, the diverse sources of contamination include: Infection at the site of primary production (*e.g*., parent stock, incubator, and farm); cross-contamination in the handling of food or byproducts; and the consumption of undercooked poultry meat, eggs, or egg products. All of these sources have been related to infection by *Salmonella* in humans [28-33]. Cases of salmonellosis have been caused by antibiotic-resistant strains of *Salmonella*, which are selected for by the indiscriminate use of antibiotics in human and veterinary medicine and in animal production [18].

**Figure-1:**
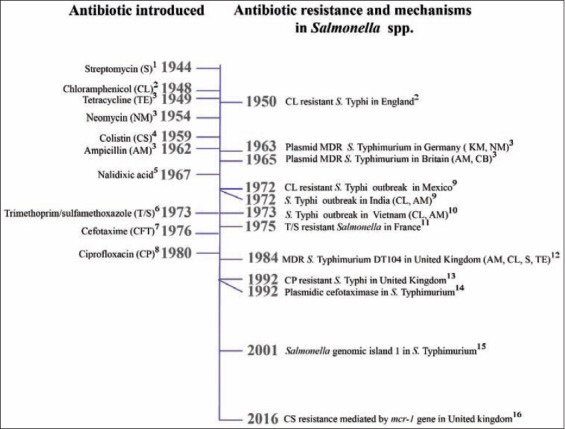
Timeline representing both antibiotic deployment and antibiotic resistance and mechanisms in *Salmonella* spp. Source: The authors. Murray *et al*. [35]^1^, Colquhoun and Weetch [36]^2^, Summers [43]^3^, Ross *et al*. [44]^4^, Jacoby *et al*. [45]^5^, Smith and Sensakovic [46]^6^, Batabyal [47]^7^, Ugboko and De [48]^8^, Anderson [49]^9^, Butler [50]^10^, Zaki and Karande [37]^11^, Crump *et al*. [38]^12^, Umasankar *et al*. [39]^13^, Bauernfeind *et al*. [40]^14^, Boyd *et al*. [41]^15^, Doumith *et al*. [42]^16^.

Historically, antimicrobials have been used as growth promoters since 1950, when it was discovered that small, sub-therapeutic quantities of antibiotics such as penicillin and tetracycline delivered to animals in feed, could enhance the weight of poultry, swine, and beef cattle [34]. The first report of a chloramphenicol-resistant *S*. Typhi occurred 2 years after the introduction of chloramphenicol in the market (1948), and 13 years later, the first plasmid-mediated transfer of antibiotic resistance in *S*. Typhimurium was reported in Germany. Since then, MDR strains have been isolated around the world (Figure-1) [35-50] . During this time, many serotypes of *Salmonella* spp. started to show resistance to antibiotics such as quinolones and cephalosporin, which have been the first choice antibiotics for the treatment of humans [51].

## Transmission of Antibiotic Resistance in *Salmonella*

Through billions of years of evolution, *Salmonella* has accumulated a large number of metabolic and protective mechanisms that can be mobilized in response to different external aggressions, including antibiotics [52]. Antibiotic resistance can be achieved by mutations in different chromosomal loci that are a part of a core set of genes, such as genomic islands. In addition, antibiotic resistance can be acquired through exogenous resistance genes carried by mobile genetic elements that can be disseminated horizontally between bacteria [48,53,54].

Genomic islands are conserved zones found in accessory regions in the *Salmonella* genome. They are considered fundamental to the evolution of this genus, as they have conferred a number of fitness advantages on their host through virulence and multidrug resistance (MD) genes [55]. Genomic island 1 (*Salmonella* genomic island 1 [SGI-1]) harbors genes associated with MD to streptomycin, spectinomycin, sulfonamides, chloramphenicol, florfenicol, tetracyclines, and β- lactam antibiotics. In addition, these genes can be carried in mobile elements such as Class 1 integrons found in the antibiotic-resistance cluster located at the 3’end of the island [56,57]. SGI-1 has been associated with the MDR strain DT 104, widely isolated from humans and food-producing animals in most parts of the world since 1980 [58].

Furthermore, *Salmonella* can acquire resistance through mobile elements such as plasmids that account for the high rates of transfer of genes that are beneficial to the survival of the host bacteria [59]. These plasmids typically encode both virulence factors and MD traits similar to those on genomic islands. However, plasmids can be transferred horizontally and may carry other mobile elements such as transposons and integrons. Therefore, they increase phenotypic diversity and confer fitness advantages during times of environmental changes, thus providing the host with opportunities for niche expansion [57,60]. Plasmids are classified according to incompatibility (Inc) types that are based on the degree of relatedness between plasmids and that control the replication of different types of plasmids within the same bacteria [61]. As a result, certain replicon types such as IncC and IncA are associated with MD and disseminate, among others, the extended-spectrum β-lactamase trait from foodborne *Salmonella* that has been responsible for past disease outbreaks [56,61,62].

Integrons are natural recombination systems that constitute one of the most efficient mechanisms for accumulating antimicrobial resistance. Their structure incorporates several open reading frames in the form of gene cassettes that code for traits related to antimicrobial resistance [57,63]. These mobile elements are composed of three key parts: A gene encoding an integrase (*intI*), a primary recombination site (*attI*), and a promoter for the transcription of captured genes (Pc) [64]. Integrons are important mainly because they are responsible for MD; some antibiotic resistance determinants are preferentially associated with integrons and include those responsible for resistance to streptomycin, trimethoprim, sulfafurazole, and some aminoglycosides [60]. Class 1 integrons have been found extensively both clinically and in the food of animal origin, and they have been associated significantly with the presence of MD. While integrons themselves are not mobile, they can be associated with a mobile element called a transposon that is capable of moving from one carrier replicon to another. Transposons are generally located on plasmids, further enhancing the spread of gene cassettes [64]. The association of integrons with mobile elements and resistance genes has led to their rapid dispersal among various bacteria found in environments exposed to antibiotics [65].

## Mechanisms of Resistance in *Salmonella*

**Figure-2:**
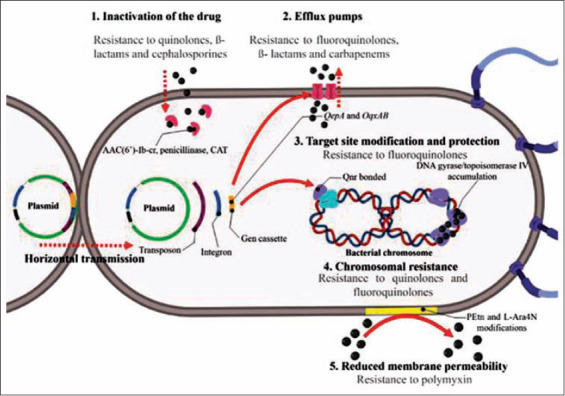
Antibiotic resistance and transmission mechanisms in *Salmonella* spp. and the respective antimicrobials which become ineffective. Source: The authors. Based on Jacoby *et al*. [45], Ugboko and De [48], Silva *et al*. [57], Munita and Arias [63], Rodríguez-Martínez *et al*. [67], Davies and Davies [66].

Antimicrobial resistance in *Salmonella* is media- ted by several mechanisms (Figure-2) [45,48,57,63,66,67]; that include drug inactivation, which is the most common cause of resistance. In this mechanism, antimicrobial agents are destroyed or inactivated through chemical modification using enzymes that catalyze reactions such as acetylation, phosphorylation, and adenylation [63]. For example, aminoglycoside-modifying acetyltransferase AAC(6’)-Ib-cr harbors two amino acid substitutions at Trp102Arg and Asp179Tyr, which confers the ability to acetylate the unsubstituted nitrogen of the C7 piperazine ring present in several quinolones [68]. In addition, enzymes such as penicillinase and chloramphenicol acetyltransferase are able to acetylate the ß-lactam ring of penicillin and some cephalosporins and the two hydroxyl groups of chloramphenicol, respectively [63,69].

*Salmonella* can acquire antibiotic resistance by protecting the target site of the antibiotic, which can either be an enzyme or a specific cell structure. For example, the plasmid-encoded quinolone resistance protein (Qnr) confers resistance to quinolones by acting as a DNA homolog that competes for the binding of DNA gyrase and topoisomerase IV [63]. This reduces the possibility of the quinolone molecule binding to DNA gyrase, thus protecting the bacteria from the lethal effects of the antibiotic [45,67]. In addition, *Salmonella* can modify the antibiotic target site to avoid it from binding. For example, the resistance of rifampicin is based on single-step point mutations that result in amino acid substitutions in the *rpoB* gene. These substitutions decrease the affinity of the drug for DNA-dependent RNA polymerase, thus allowing the transcription of *rpoB* to continue [63,70].

Another mechanism of resistance in *Salmonella* is through the reduction in its membrane permeability, thus preventing drug entry [48]. The alteration occurs when membrane proteins undergo changes through new genetic information that alters the membrane transport system pores and thus prevents the passage of antibiotics. In the case of polymyxin resistance, a modification in the lipid A moiety of the lipopolysaccharide structure consisting of phosphoethanolamine and 4-amino-4-deoxy-L-arabinose results in a reduction in the net negative charge of the membrane, which reduces its affinity for polymyxin [71,72].

*Salmonella* has also developed the ability to pump out a drug after it has gained entry into the system, using efflux pumps or MD pumps. This mechanism is relatively nonspecific and can pump out many different drugs, including fluoroquinolones, ß- lactams, and carbapenems [63]. Many of these efflux pumps are encoded by genes within mobile elements such as plasmids. For example, genes such as *QepA* and *oqxAB* confer resistance to several fluoroquinolones [68].

In *Salmonella* as well as in other bacteria, resistance to antibiotics may be mediated to a lesser extent by chromosomal mechanisms based on mutations in genes that code for either the target of the drug or the membrane transport system that controls the uptake of the drug [48]. For example, chromosomal-mediated quinolone resistance may result from selective pressure on the bacterial population due to the uncontrolled use of the drug. Under normal conditions, quinolones enter bacteria through porins and then bind to the gyrase/topoisomerase IV–DNA complex. The resulting bound complex is prevented from replicating, thus explaining the drug’s bacteriostatic action. *Salmonella* has developed single point mutations in the quinolone resistance determining region of the topoisomerase genes *parC* and *parE* and the DNA gyrase genes *gyrA* and *gyrB*. As a result, the lethal action of some fluoroquinolones and quinolones is blocked by the accumulation of these genes and by the structural change in the protein that leads to a reduced affinity for the drug [37,73].

## Antibiotic Resistance by the Family in Poultry and Poultry Products

Our literature search used the search terms “*Salmonella*” in combination with “chicken,” “broiler,” “raw,” “poultry,” “eggs,” and “antibiotic resistance.” We selected a total of 112 papers published between 2003 and September 2019.

We developed resistance profiles from production segments worldwide consisting of broiler chickens, chicken at processing plants, markets, eggs, and egg-laying hens. The choice of antimicrobials is based on reports of the WHO, namely, the List of Critically Important Antimicrobials from Human Medicine [11] and the List of Essential Medicines [25]. We took into account five of the most important antibiotic families consisting of aminoglycosides, β- lactams, quinolones and fluoroquinolones, cephalosporins, and carbapenems and monobactam. Antibiotic resistance prevalence data were summarized according to the antimicrobial, using the median and IQR across studies. The data were plotted for comparative purposes in heat maps generated by GraphPad Prism software v.7 (La Jolla, California). The prevalence of resistance against specific antimicrobials in individual studies was compiled in tabular form and antibiotic resistance was classified as either high (>50%), medium (21-50%), or low (0-20%). We considered isolates from the studies as MDR if they showed resistance to three or more antimicrobials.

## Antibiotic Resistance in Poultry Farms Worldwide

**Figure-3:**
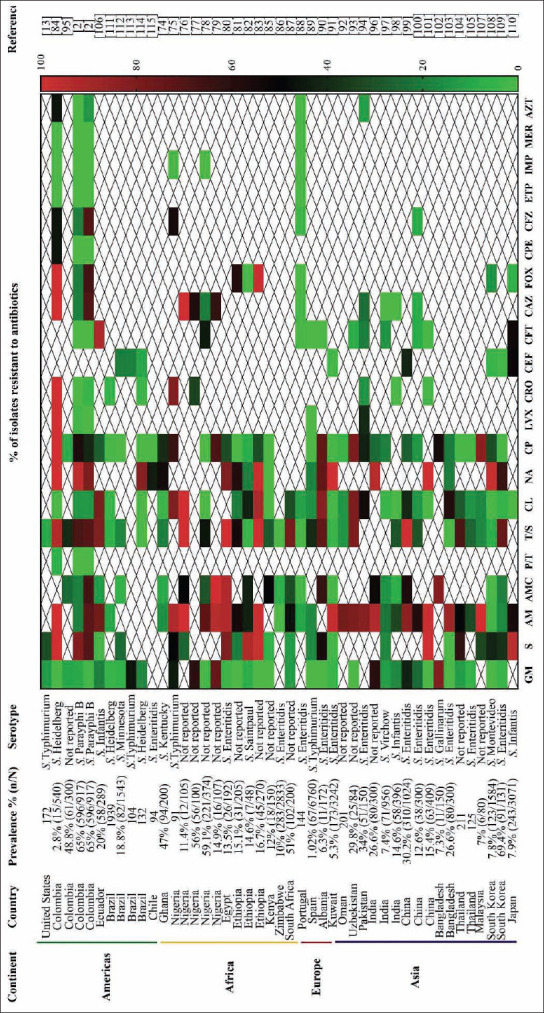
Antibiotic resistance patterns in *Salmonella* spp. isolated from poultry farms in the world. ^1^Aminoglycosides: Gentamycin (GM), streptomycin (S); ^2^β-lactams: Ampicillin (AM), amoxicillin/clavulanic acid (AMC), piperacillin/tazobactam (P/T); ^3^folate antagonist: Trimethoprim/sulfamethoxazole (T/S); ^4^phenicol: Chloramphenicol (CL); ^5^quinolones and fluoroquinolones: Nalidixic acid (NA), ciprofloxacin (CP), levofloxacin (LVX); ^6^cephalosporines: Ceftriaxone (CRO), ceftiofur (CEF), cefotaxime (CFT), ceftazidime (CAZ), cefoxitin (FOX), cefepime (CPE), cefazolin (CFZ); ^7^carbapenems: Ertapenem (ETP), imipenem (IMP), meropenem (MER); ^8^monobactams: Aztreonam (AZT) [2,13,74-115].

A total of 45 publications investigated antibiotic resistance in 7033 isolates from raw chicken meat from the Americas (11 studies), Africa (13 studies), Europe (3 studies), and Asia (18 studies) (Figure-3) [2,13,74-115]. The median prevalence of *Salmonella* in broiler chickens was 40.5% (IQR 11.5-58.2%), and the most prevalent serotypes were *S*. Enteritidis (13 studies) and *S*. Typhimurium (4 studies), while the presence of *S*. Heidelberg (3 studies) and *S*. Infantis (3 studies) was also found. Thirteen of the articles did not report *Salmonella* serotypes.

MD was found in 91.1% (41/45) of the articles, representing research conducted worldwide. High levels of antibiotic resistance (80.3% and 64.8%, respectively) were found in *Salmonella* isolates from broiler chickens treated by commonly used antibiotics in poultry production, such as nalidixic acid (fluoroquinolone family; IQR 43.6-97.6%) and ampicillin (β-lactam family; IQR 17.7-92.1%). Medium levels of resistance (33%, 29.4%, and 39.3%, respectively) were found to antibiotics such as streptomycin (aminoglycosides; IQR 16.4-80.8%), amoxicillin/clavulanic acid (β-lactam family; IQR 9-56.7%), and trimethoprim/sulfamethoxazole (folate antagonist; IQR 7.9-76.5%). Surprisingly, resistance levels were low (6%) to gentamycin (IQR 0-17.1%) although it is widely used in day-old chickens [116]. Similarly, resistance levels were low (19%) to ciprofloxacin (IQR 0.6-40.1%), an antibiotic broadly used in poultry production. Resistance levels were also low (13.6%) against chloramphenicol (IQR 0.3-38.2%), which is probably due to the low usage of this antibiotic in recent years due to its various toxic and carcinogenic effects in humans [117].

In the cephalosporin family, antibiotic resistance levels were low (4.1%, 7.9%, 18.8%, 0%, and 18.6%, respectively) for ceftriaxone (IQR 0-28.9%), cefotaxime (IQR 0-20%), ceftazidime (IQR 1.3-77.2%), cefepime (IQR 0-46%), and cefazolin (IQR 5.3-84.8%); while the resistance level was medium (38.3%) for cefoxitin (IQR 4.2-90%). It should be mentioned that ceftiofur resistance was low (9.8%), despite being used in day-old chickens in poultry production (IQR 2.2-20%) [116]. In addition, no resistance was detected for the carbapenem family (ertapenem, imipenem, and meropenem).

Levels of antibiotic-resistant *Salmonella* in European countries and the US tended to be low, which is related to the establishment of control and monitoring programs such as the European Food Safety Authority and the European Centre for Disease Prevention and Control, and the National Antibiotic Resistance Monitoring System in the US [118].

## Antibiotic Resistance in Raw Chicken Meat at Processing Plants and Markets

**Figure-4:**
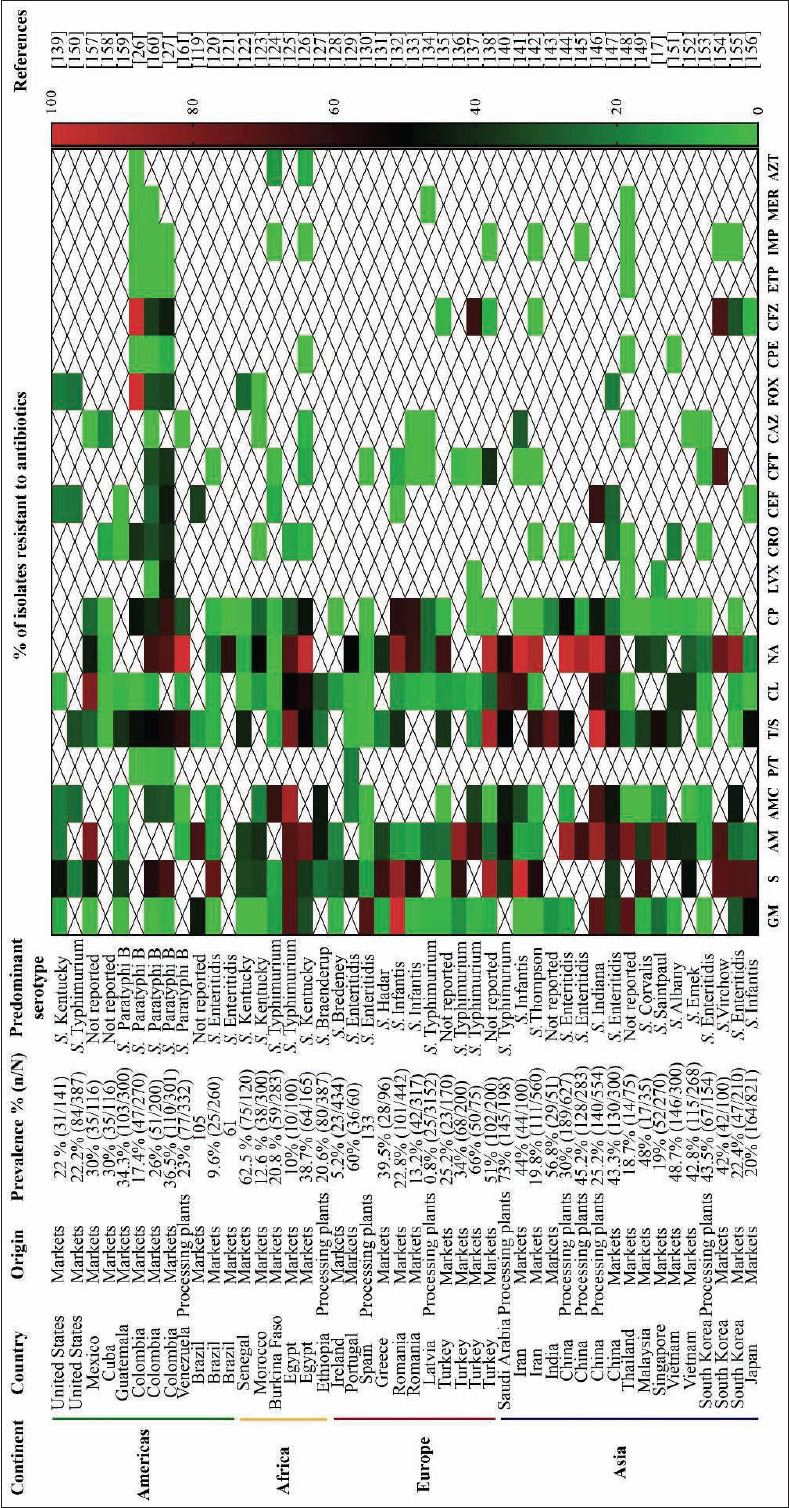
Antibiotic resistance patterns in *Salmonella* spp. isolated from chickens in processing plants and at market in the world. ^1^Aminoglycosides: Gentamycin (GM), streptomycin (S); ^2^β-lactams: Ampicillin (AM), amoxicillin/ clavulanic acid (AMC), piperacillin/tazobactam (P/T); ^3^folate antagonist: Trimethoprim/sulfamethoxazole (T/S); ^4^phenicol: Chloramphenicol (CL); ^5^quinolones and fluoroquinolones: Nalidixic acid (NA), ciprofloxacin (CP), levofloxacin (LVX); ^6^cephalosporines: Ceftriaxone (CRO), ceftiofur (CEF), cefotaxime (CFT), ceftazidime (CAZ), cefoxitin (FOX), cefepime (CPE), cefazolin (CFZ); ^7^carbapenems: Ertapenem (ETP), imipenem (IMP), meropenem (MER); ^8^monobactams: Aztreonam (AZT) [17,26,27,119-161].

A total of 46 publication investigated antibiotic resistance in 3301 isolates from raw chicken meat in study sites in the Americas (12 studies), Africa (6 studies), Europe (11 studies), and Asia (17 studies) (Figure-4) [17,26,27,119-161]. It should be mentioned that most of the studies focused on antibiotic resistance at markets (37 studies) rather than processing plants (9 studies). This is probably because markets are the last segment in the poultry production chain and are thus widely linked to human infections.

The median prevalence of *Salmonella* in raw chicken meat was 30% (IQR 20-43.5%), and the most prevalent serotypes were *S*. Enteritidis (9 studies) and *S*. Typhimurium (7 studies). These trends are similar to those reported in broilers, eggs, and egg-laying hens. In addition, the serotypes *S*. Paratyphi B (5 studies), *S*. Infantis, and *S*. Kentucky (4 studies) have also been reported. Only seven articles did not report the most prevalent serotype in their studies.

MD was present in 97.8% (45/46) of the articles included in this study. A high level of antibiotic resistance (60.7%) in *Salmonella* isolates was associated with commonly used antibiotics in poultry production, such as nalidixic acid (fluoroquinolone family; IQR 26.8-86.6%). Medium levels of antibiotic resistance (47.3%, 35.5%, and 37.9%, respectively) were associated with antibiotics such as streptomycin (aminoglycoside family; IQR 34.2-69.2%), ampicillin (β-lactam family; IQR 14.9-68%), and trimethoprim/sulfamethoxazole (folate antagonist family; IQR 16-54.2%).

Low levels of resistance (7%, 5%, and 8%, respectively) were associated with gentamycin (IQR 1.1-31%), ciprofloxacin (IQR 0-30%), and chloramphenicol (IQR 3.6-37.3%), findings that are similar to corresponding antibiotics in broiler chickens (above). It should be mentioned that antibiotics commonly used in human medicine are poorly represented in the studies included in this review. However, in most cases, *Salmonella* isolates showed low levels of resistance to these antibiotics. Isolates from raw chicken meat showed medium levels of resistance (21.4%, 23.5%, and 32.6%, respectively) to antibiotics from the cephalosporin family such as ceftiofur (IQR 2-35%), cefoxitin (IQR 19.1-32.7%), and cefazolin (IQR 5.5-65.2%), while low levels of resistance (0%, 8%, 2.2%, and 0%, respectively) were found for ceftazidime (IQR 0-4.7%), ceftriaxone (IQR 0-25.4%), cefotaxime (IQR 0-26%), and cefepime (IQR 0-1.9%). This family is considered critically important for clinical treatment because it has an extended spectrum of effectivity and can be safely administered to pregnant women and children [162]. No resistance has been detected against the carbapenem family of antibiotics such as ertapenem, imipenem and meropenem, which is consistent among broiler chickens, eggs, and egg-laying hens.

## Antibiotic Resistance in Eggs and Egg-laying Hens in the World

**Figure-5:**
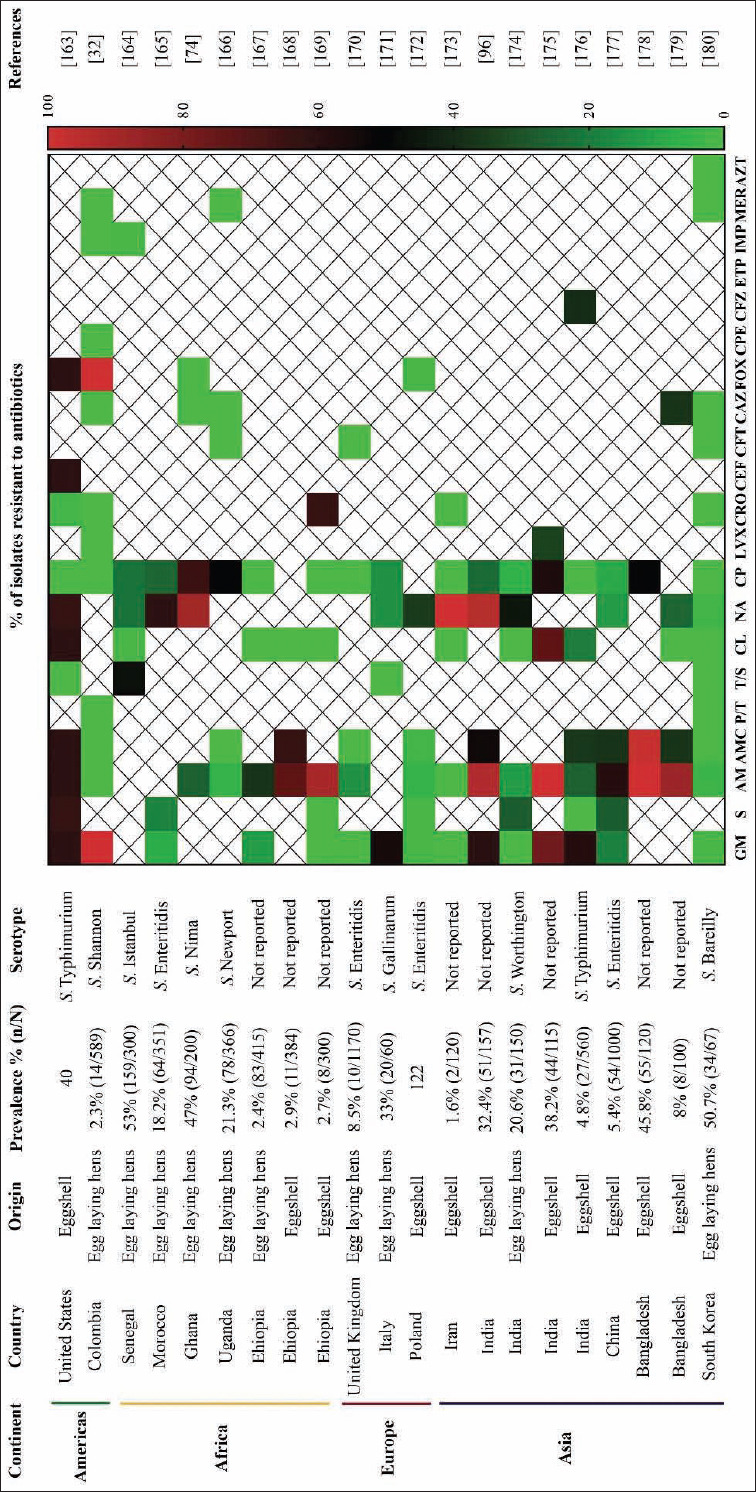
Antibiotic resistance patterns in *Salmonella* spp. isolated from egg laying hen farms and eggs in different parts of the world. ^1^Aminoglycosides: Gentamycin (GM), streptomycin (S); ^2^β-lactams: Ampicillin (AM), amoxicillin/clavulanic acid (AMC), piperacillin/tazobactam (P/T); ^3^folate antagonist: Trimethoprim/sulfamethoxazole (T/S); ^4^phenicol: Chloramphenicol (CL); ^5^quinolones and fluoroquinolones: Nalidixic acid (NA), ciprofloxacin (CP), levofloxacin (LVX); ^6^cephalosporines: Ceftriaxone (CRO), ceftiofur (CEF), cefotaxime (CFT), ceftazidime (CAZ), cefoxitin (FOX), cefepime (CPE), cefazolin (CFZ); ^7^carbapenems: Ertapenem (ETP), imipenem (IMP), meropenem (MER); ^8^monobactams: Aztreonam (AZT) [32,74,96,163-180].

A total of 21 publications investigated antibiotic resistance in 1009 isolates from egg-laying hens farms and eggs from Asia (9 studies), Africa (7 studies), Europe (3 studies), and the Americas (2 studies) (Figure-5) [32,74,96,163-180]. Most of the studies report the presence of *Salmonella* on eggshells (11 studies), followed by egg-laying hens (10 studies). The median prevalence of *Salmonella* in eggs and egg-laying hens was 40% (IQR 14.2-51.5%), and the most prevalent serotype was *S*. Enteritidis (4 studies), followed by *S*. Typhimurium (2 studies). Eight studies did not report serotypes. The high prevalence of *S*. Enteritidis in eggs and egg-laying hens is due to the ability of this serotype to disseminate in reproductive tissues such as the ovary and oviduct in infected hens [181]. As a result, this serotype is often reported to infect humans through the consumption of eggs, which is an important transmission source [182].

Medium levels of antibiotic resistance (31.9%, 36.9%, and 40.3%, respectively) were found in *Salmonella* strains isolated from eggs and laying hens for ampicillin (β-lactam family; IQR 5-88.1%), amoxicillin/clavulanic acid (β-lactam family; IQR 0-58.9%), and nalidixic acid (fluoroquinolone family; IQR 167-82.9%). Low levels of antibiotic resistance (12.5%, 18%, and 7.6%, respectively) were found for aminoglycoside antibiotics such as gentamycin (IQR 0-60.7%) and streptomycin (IQR 0-27.7%) and for the fluoroquinolone ciprofloxacin (IQR 0-31%).

No resistance has been detected to antibiotics commonly used in human medicine that is of “highest priority critically important antimicrobials” according to the WHO [11]. These include cephalosporins such as ceftriaxone, cefotaxime, ceftazidime, and cefepime; and carbapenems such as imipenem, meropenem, and aztreonam.

Low levels of antibiotic resistance (12.5%, 18%, 0%, 7.6%, and 0%, respectively) were found for aminoglycosides such as gentamycin (IQR 0-60.7%) and streptomycin (IQR 0-27.7%); and for chloramphenicol (IQR 0-19.9%), ciprofloxacin (IQR 0-31%), and trimethoprim/sulfamethoxazole (IQR 0-35.2%). In the case of trimethoprim/sulfamethoxazole, the low level of resistance is probably because sulfonamides are not commonly used in egg-laying hens, in contrast to broiler chickens [116].

## Conclusion

Antibiotic resistance is a global health threat that impacts the poultry industry, as MDR *Salmonella* strains have been reported mainly originating from poultry sources. This study found a worldwide median prevalence values of *Salmonella* in broiler chickens of 40.5%, in raw chicken meat of 30% and in eggs and egg-laying hens of 40.5% as well as a higher number of MDR isolates from poultry farms (91.1%) and from raw chicken meat at processing plants and markets (97.8%) worldwide. In addition, the highest antibiotic resistance levels within the poultry production chain were found for nalidixic acid and ampicillin. This situation demands a better understanding of the bacterial resistance in animal and human isolates, to enable the establishment of control strategies that reduce the risk of antibiotic-resistant pathogens. Government entities, researchers, and poultry producers have the responsibility of managing antibiotic resistance by reducing the use of antibiotics, conducting active surveillance of MDR strains, and searching for alternatives to control and prevent infectious disease outbreaks.

## Authors’ Contributions

REC, MPH, RR, and ISR conceptualized the review. REC, MPH, and RR collected the literature. REC made the figures. REC and MPH made the statistical analysis. All authors were involved in the writing, analysis of the data, and reviewed the manuscript, and they approved the final manuscript.
